# Probiotic and Antimicrobial Potential of Endophytic *B. subtilis UzMU25* Isolated from *Inula helenium*

**DOI:** 10.3390/microorganisms14081819

**Published:** 2026-08-18

**Authors:** Luiza Tagayeva, Kunduz Normurodova, Shermat Jabborov, Bobur Khasanov, Jamoliddin Razzokov

**Affiliations:** 1 Department of Biotechnology and Microbiology, Faculty of Biology and Ecology, National University of Uzbekistan, University Street 4, Tashkent 100174, Uzbekistan; tagaevaluiza646@gmail.com (L.T.); normurodovak@gmail.com (K.N.); 2 Scientific Research Institute of Livestock and Poultry, Tashkent, Kibray District, Qizil Shalola Village, Tashkent 111121, Uzbekistan; boburxasanov.97@mail.ru; 3 Institute of Fundamental and Applied Research, National Research University TIIAME, Tashkent 100000, Uzbekistan; jrazzokov@gmail.com; 4 Department of Natural Sciences, Karshi State Technical University, Mustaqillik Avenue Street 225, Karshi 180100, Uzbekistan; 5 Department of Biotechnology, Tashkent State Technical University, Universitet 2, Tashkent 100095, Uzbekistan

**Keywords:** *Inula helenium*, endophytes, *B. subtilis UzMU25*, probiotic properties, antagonistic activity, veterinary biotechnology

## Abstract

Endophytic bacteria associated with medicinal plants represent a potential source of microorganisms with useful enzymatic and antagonistic properties. In this study, 14 bacterial isolates were recovered from the leaves, roots, flowers, and petioles of *Inula helenium* and screened for morphological, biochemical, and hydrolytic characteristics. Isolate IH-B3, which showed the most pronounced starch- and casein-hydrolysis zones during preliminary screening, was selected for further characterization. MALDI-TOF mass spectrometry assigned the isolate to *Bacillus subtilis* with an identification score of 2.26, and the strain was designated *B. subtilis* UzMU25. Its 1444 bp 16S rRNA gene sequence was deposited in GenBank under accession number PZ593820. The strain was catalase- and lecithinase-positive but gelatinase- and hemolysis-negative, and it grew at pH 6.5 and in the presence of horse bile under the qualitative assay conditions used. Hydrolysis-zone diameters ranged from 46 to 52 mm for amylase, 34 to 42 mm for protease, and 10 to 16 mm for lipase activity. In direct antagonism assays, UzMU25 inhibited all six bacterial and fungal test organisms, producing inhibition zones of 26–36 mm. Biofilm biomass varied with incubation time and reached its highest corrected OD_590_ value at 12 h (0.780 ± 0.025). Antibiotic-disc testing showed a 10 mm inhibition zone for vancomycin, indicating reduced susceptibility under the applied conditions and requiring further investigation of its genetic basis and potential transferability. No mortality was observed during the preliminary acute oral study following administration of the tested preparation at doses up to 10,000 mg kg^−1^, and no visible dermal or conjunctival irritation was detected. Overall, UzMU25 exhibited preliminary enzymatic, antagonistic, and biofilm-forming characteristics of biotechnological interest. However, whole-genome antimicrobial-resistance screening and more comprehensive phenotypic and toxicological evaluations are required before the strain can be recommended for probiotic or other practical applications.

## 1. Introduction

Endophytic microorganisms inhabit the internal tissues of plants without causing disease symptoms and play important roles in plant growth, health, and adaptation to environmental stresses [1,2]. Among them, endophytic bacteria have attracted considerable attention because of their ability to produce bioactive metabolites, promote host development and suppress pathogenic microorganisms [3,4,5]. Medicinal plants are considered particularly valuable reservoirs of beneficial endophytes due to their rich phytochemical composition and unique ecological niches [6,7]. These microorganisms are increasingly being investigated as potential sources of novel probiotics, antimicrobial agents and biotechnological products for agricultural, veterinary and pharmaceutical applications [8,9,10,11].

Among beneficial bacteria, members of the genus *Bacillus* have gained significant interest because of their spore-forming ability, environmental stability and broad spectrum of biological activities [12,13]. Several *Bacillus* species are widely used as probiotics in animal husbandry, aquaculture and agriculture due to their ability to survive gastrointestinal conditions, produce extracellular enzymes and synthesize antimicrobial metabolites [14,15]. In particular, *Bacillus subtilis* is recognized for producing hydrolytic enzymes such as amylases, proteases, and lipases, which enhance nutrient utilization and support digestive processes [16]. Furthermore, *B. subtilis* strains can inhibit the growth of bacterial and fungal pathogens through the production of lipopeptides, bacteriocins, and other secondary metabolites, while biofilm formation contributes to their persistence and colonization efficiency [17,18].

*Inula helenium* L. (elecampane) is a medicinal plant traditionally used for the management of respiratory and gastrointestinal disorders and is known to contain several classes of biologically active compounds, including sesquiterpene lactones, flavonoids, and phenolic constituents [19,20,21]. Although its phytochemical and pharmacological properties have been widely investigated, considerably less information is available on its cultivable endophytic bacterial community. In particular, phenotypic data concerning the extracellular enzyme production, direct antagonistic activity, biofilm formation, antibiotic susceptibility, and preliminary safety characteristics of *Bacillus* isolates recovered specifically from *I. helenium* remain limited. Characterization of such isolates may therefore expand current knowledge of medicinal-plant-associated endophytes and identify strains of potential biotechnological interest [22,23,24].

The evaluation of a candidate probiotic strain requires consideration of both functional properties and potential safety concerns. Desirable characteristics may include extracellular enzyme production, antagonistic activity, tolerance to selected environmental stresses, and the ability to form attached biomass. However, these traits alone are insufficient to establish probiotic safety. Hemolytic and tissue-degrading activities, antibiotic-susceptibility profiles, the possible presence of acquired or transferable resistance determinants, and in vivo tolerability must also be assessed. For spore-forming *Bacillus* strains, phenotypic observations should ultimately be complemented by genome-based screening for antimicrobial-resistance and virulence-associated determinants before practical application is considered.

The present study aimed to isolate and characterize cultivable endophytic bacteria from different tissues of *I. helenium* and to investigate the biological properties of the selected isolate, designated *Bacillus subtilis* UzMU25. The strain was identified using MALDI-TOF mass spectrometry and 16S rRNA gene sequencing and was evaluated for morphological and biochemical characteristics, extracellular hydrolytic enzyme activities, qualitative tolerance to pH 6.5 and horse bile, antibiotic susceptibility, direct antagonistic activity against bacterial and fungal test organisms, and biofilm formation. Preliminary in vivo tolerability, cumulative toxicity, and local irritation were also examined under the applied experimental conditions. The study was intended to provide an initial phenotypic characterization of UzMU25 and to determine whether the strain merits further molecular, mechanistic, and comprehensive safety evaluation, rather than to establish its immediate suitability for probiotic or commercial use.

## 2. Materials and Methods

### 2.1. Isolation of Endophytic Bacteria

The medicinal plant *Inula helenium* L. was used as the source of endophytic bacteria. Plant tissues including leaves, roots, flowers, and petioles were collected and processed under sterile conditions. Endophytic bacteria were isolated according to standard microbiological procedures following surface sterilization of plant tissues. A total of 14 bacterial isolates were recovered and screened based on their cultural, morphological, and enzymatic characteristics. Five active isolates were selected for further evaluation. Among them, one isolate exhibited superior hydrolytic activity and was subsequently identified as *B. subtilis UzMU25*.

*Inula helenium* L. is a perennial herbaceous medicinal plant belonging to the family Asteraceae and the order Asterales. It is naturally distributed across southeastern Europe, the Caucasus, and western and Central Asia, including Uzbekistan. The plant typically grows in fertile, organic-matter-rich soils in mountainous and foothill regions. Healthy *I. helenium* plants showing no visible symptoms of disease were collected during April and May from the foothill areas of Bostanlyk District, Tashkent Region, Uzbekistan. Leaves, stems, roots, flowers, and petioles were transferred to the laboratory and processed under aseptic conditions.

Endophytic microorganisms were isolated using a surface-sterilization procedure adapted from Strobel et al. [25]. Plant samples were initially washed under running tap water for 10–15 min to remove adhering soil particles, dust, and other surface debris, followed by three rinses with sterile distilled water. The tissues were then immersed in 70% ethanol for 1 min and subsequently treated with 2% sodium hypochlorite. Exposure time to sodium hypochlorite was adjusted according to tissue structure: relatively delicate leaf tissues were treated for approximately 2 min, whereas stems and roots were exposed for up to 5 min. After surface sterilization, the samples were rinsed four to five times with sterile distilled water to remove residual disinfectants.

The effectiveness of surface sterilization was assessed by plating 100 µL of the final rinse water onto nutrient agar. The control plates were incubated under the same conditions used for bacterial isolation. The absence of microbial growth on these plates was considered evidence that cultivable surface-associated microorganisms had been effectively removed.

Surface-sterilized plant tissues were cut into approximately 1–2 cm segments using a sterile scalpel. The tissue segments were placed on nutrient agar for bacterial isolation and on potato dextrose agar for the recovery of fungal endophytes. Bacterial cultures were incubated at 30–37 °C, whereas fungal cultures were incubated at 25–28 °C. Plates were monitored for 3–14 days, depending on the growth rate of the emerging microorganisms.

Colonies emerging from the internal plant tissues were selected based on visible morphological differences, including colony shape, pigmentation, margin characteristics, surface texture, transparency, elevation, glossiness, and pigment production in the culture medium. Morphologically distinct colonies were repeatedly subcultured on fresh agar media until pure cultures were obtained. The purified isolates were maintained under appropriate storage conditions for subsequent morphological, biochemical, enzymatic, and biological characterization.

### 2.2. Morphological, Biochemical and Physiological Characterization

Morphological and cultural characteristics of the selected isolates were assessed on nutrient agar media. The probiotic-related properties of *B. subtilis* UzMU25 were evaluated through biochemical and physiological tests, including carbohydrate utilization, enzyme production, growth at different temperatures, tolerance to acidic conditions, and resistance to bile salts. Growth was evaluated on MRS medium following incubation at 10 °C and 45 °C for periods ranging from 48 h to 10 days.

Catalase activity was determined by applying a drop of 10% hydrogen peroxide to a bacterial cell suspension. Immediate formation of oxygen bubbles was considered a positive reaction. Acidity production was evaluated using sterile skimmed milk. After incubation for 24 h at 37 °C, titratable acidity was determined by titration with 0.1 N NaOH using phenolphthalein as an indicator and expressed in Turner degrees (°T). Tolerance to bile salts was assessed using a solution containing pancreatin (0.1 g), horse bile (0.3 g), sterile 0.5% NaCl solution, and 0.1 N NaOH for pH adjustment. The solution was sterilized by filtration through a 0.22 μm membrane filter before use.

### 2.3. Determination of Lecithinase, Gelatinase and Hemolytic Activities

Lecithinase activity was evaluated in meat-peptone broth supplemented with egg yolk. Following incubation at 37 °C for 24 h, the appearance of turbidity and suspended precipitates was recorded as a positive reaction.

Gelatinase activity was determined using nutrient medium supplemented with 15% gelatin. The bacterial culture was inoculated by the stab method and incubated at 37 °C for 24–48 h. Gelatin liquefaction was recorded as evidence of gelatinase production.

Hemolytic activity was investigated on meat-peptone agar supplemented with 5% defibrinated blood. Plates were incubated at 37 °C for 24–48 h and subsequently refrigerated for 18–24 h to enhance visualization of hemolytic zones.

Catalase activity was determined by applying one drop of 10% hydrogen peroxide to a fresh bacterial colony or cell suspension. Immediate formation of visible gas bubbles, resulting from the release of molecular oxygen, was recorded as a positive catalase reaction [26,27].

Lecithinase activity was assessed using egg-yolk-supplemented meat-peptone medium. One-quarter of a fresh egg yolk was aseptically added to 100 mL of sterile meat-peptone broth and mixed thoroughly. The medium was dispensed into sterile tubes in 4–5 mL aliquots. Uninoculated tubes were incubated at 37 °C for 24 h to confirm sterility. The test strain was then inoculated into the medium and incubated at 37 °C for 24 h. The appearance of marked turbidity or a white precipitate in the inoculated medium was considered a positive lecithinase reaction, whereas the absence of these changes was recorded as negative. The assay was performed according to MUK 4.2.2602-10 [28].

Hemolytic activity was evaluated on meat-peptone agar supplemented with 5% defibrinated horse blood. The blood was aseptically added to the sterilized agar medium after cooling to 48–50 °C. The medium was mixed carefully, poured into sterile Petri dishes, and allowed to solidify. Uninoculated plates were incubated at 37 ± 1 °C for 24 h to confirm sterility. The test strain was subsequently streak-inoculated onto the blood agar plates and incubated at 37 ± 1 °C for 24–48 h. Following incubation, the plates were maintained at 4 °C for an additional 18–24 h to improve visualization of possible hemolytic reactions. The presence of a visible hemolysis zone around bacterial growth was recorded as a positive reaction, whereas the absence of hemolysis was considered a negative result, according to MUK 4.2.2602-10.

Gelatinase activity was determined using meat-peptone broth containing 12% gelatin. The medium was dispensed into sterile tubes in 4–5 mL aliquots. A single colony of the test strain was inoculated into the gelatin-containing medium using the stab-inoculation method. Uninoculated medium was included as a negative control. The inoculated and control tubes were incubated at 37 °C for 24–48 h and subsequently cooled at 4 °C for 20–30 min. Gelatin liquefaction that persisted after cooling was recorded as a positive gelatinase reaction, whereas solidification of the medium was interpreted as a negative result. The intensity of bacterial growth and the extent of gelatin liquefaction were recorded according to MUK 4.2.2602-10.

### 2.4. Determination of Hydrolytic Enzyme Activities

Amylase activity was evaluated on a solid medium containing peptone (10 g), soluble starch (2 g), and KH_2_PO_4_ (5 g). The medium was dispensed into sterile Petri dishes in 20 mL portions. A freshly activated culture of the test strain was streak-inoculated onto the medium and incubated at 37 °C for 48 h. After incubation, 5 mL of Lugol’s iodine solution was applied to the surface of each plate. Iodine forms a blue complex with non-hydrolysed starch, whereas starch degradation by extracellular amylase produces a clear zone around bacterial growth. The diameter of the starch-hydrolysis zone was measured in millimetres according to MUK 4.2.2602-10.

Lipase activity was assessed using an agar medium containing Tween 80, peptone (10 g), NaCl (5 g), CaCl_2_·H_2_O (1 g), and agar (18 g). The prepared medium was dispensed into sterile Petri dishes in 20 mL portions, and the freshly activated test strain was streak-inoculated onto the agar surface. The plates were incubated under the specified culture conditions and examined for 2–5 days. Lipase activity was evaluated by measuring the diameter of the visible hydrolysis or precipitation zone formed around the bacterial growth.

Protease activity was evaluated using a milk-containing medium prepared with 3% aqueous agar. Aliquots of 20 mL were poured into sterile Petri dishes, and the freshly activated test strain was streak-inoculated onto the medium. The plates were incubated for 2–10 days. Following incubation, 5% trichloroacetic acid was applied to the agar surface, and the resulting casein-hydrolysis zones were measured and analysed [12].

Tolerance of the test strain to gastrointestinal-stress-related conditions was initially assessed by examining growth at different pH values and in the presence of bile.

For the bile-tolerance assay, MRS medium was supplemented with 0.3 g of horse bile and dispensed into sterile Petri dishes in 20 mL portions. The test strain was streak-inoculated onto the bile-containing medium. The same medium prepared without horse bile and inoculated with the test strain served as the untreated growth control. The plates were incubated at 37 °C for 24 h. Growth on the bile-containing medium was compared with growth on the control medium, and isolates showing visible growth in the presence of horse bile were considered bile tolerant under the applied qualitative assay conditions.

### 2.5. Identification of the Selected Isolate

The isolate exhibiting the highest hydrolytic activity was initially identified using matrix-assisted laser desorption/ionization time-of-flight mass spectrometry (MALDI-TOF MS). The protein mass-spectrum profile obtained from the isolate was compared with reference spectra available in the instrument database. MALDI-TOF MS generates a characteristic mass-spectral fingerprint composed predominantly of abundant ribosomal proteins and compares this profile with validated reference spectra. The resulting score reflects the degree of similarity between the spectrum of the tested isolate and the corresponding database reference. According to the scoring criteria applied by the MALDI-TOF system, a score of ≥2.00 was considered indicative of reliable species-level identification. Based on the best database match, the isolate was assigned to *Bacillus subtilis* and was subsequently designated *B. subtilis* UzMU25.

For subsequent molecular identification, genomic DNA was extracted from an actively growing 15 h pure culture of *B. subtilis* UzMU25 using the GeneJET Genomic DNA Purification Kit according to the manufacturer’s instructions (Thermo Fisher Scientific, Vilnius, Lithuania). The nearly full-length 16S rRNA gene was amplified using the universal bacterial primer pair 27F/1492R. The DNA extraction procedure followed the GeneJET Genomic DNA Purification Kit User Guide (Cat. Nos. K0721 and K0722; Publication No. MAN0012663, Rev. C00; Thermo Fisher Scientific, Vilnius, Lithuania, 2024).

The concentration and purity of the extracted genomic DNA were assessed using a NanoDrop™ Eight Spectrophotometer (Thermo Fisher Scientific, Waltham, MA, USA) by measuring absorbance at 260 and 280 nm. DNA quality was evaluated from the corresponding absorbance ratios before PCR amplification. The nearly full-length 16S rRNA gene was amplified using the universal bacterial primer pair 27F/1492R. In addition, targeted PCR screening was performed for selected functional loci associated with extracellular protease production, antimicrobial activity, exopolysaccharide and levan biosynthesis, and cellular stress response, as well as for selected toxin-associated loci. The primer sequences and their target genes are provided in Supplementary Table S2. Detailed information on primer design and PCR screening is presented in Supplementary Materials. Molecular identification of the selected isolate was further performed by sequencing the 16S rRNA gene. The resulting sequence was compared with reference sequences available in the NCBI database, and the isolate was assigned to *Bacillus subtilis*. The 16S rRNA gene sequence of the strain, designated *B. subtilis* UzMU25, was deposited in the NCBI GenBank database under accession number PZ593820.

### 2.6. Antibiotic Susceptibility Testing

The susceptibility of *B. subtilis* UzMU25 to ciprofloxacin, vancomycin, chloramphenicol (levomycetin), imipenem, meropenem, erythromycin, clindamycin, linezolid, and norfloxacin was evaluated using the disk diffusion method on Mueller–Hinton agar. Following incubation at 37 °C for 24 h, inhibition-zone diameters were measured and interpreted according to accepted microbiological criteria.

Antibiotic susceptibility of *Bacillus subtilis* UzMU25 was evaluated using the disc-diffusion method in accordance with the procedures described in MUK 4.2.2602-10. Following incubation, the diameter of the growth-inhibition zone surrounding each antibiotic disc was measured in millimetres. Where applicable, the results were interpreted as susceptible, intermediate, or resistant according to the relevant EUCAST interpretive criteria. For antibiotic–organism combinations for which validated *Bacillus subtilis*-specific breakpoints were unavailable, inhibition-zone diameters were reported descriptively without assigning categorical susceptibility classifications.

### 2.7. Evaluation of Antagonistic Activity

The antimicrobial activity of *B. subtilis* UzMU25 was assessed against *Staphylococcus aureus*, *Pseudomonas aeruginosa*, *Escherichia coli*, *Serratia marcescens*, *Bacillus subtilis*, and *Candida albicans*. Antagonistic activity was determined using agar diffusion assays, and inhibition-zone diameters were measured after incubation. Fresh cultures of the test microorganisms were suspended in sterile saline and adjusted to a standardized inoculum concentration. The standardized suspensions were uniformly inoculated onto the surfaces of the appropriate agar media.

The UzMU25 preparation was applied to wells prepared in the inoculated agar. Sterile saline and uninoculated culture medium were included as negative controls. Appropriate antibacterial agents were used as positive controls for the bacterial test organisms, while an appropriate antifungal agent was used for *C. albicans*. Plates were incubated under organism-specific conditions, after which the diameters of the inhibition zones were measured in millimetres. All experiments were performed in three independent replicates, and the results were expressed as mean ± standard deviation.

### 2.8. Biofilm Formation Assay

Biofilm formation was quantified using the crystal violet microtiter plate assay in sterile 96-well polystyrene plates, with modifications based on [24,29]. Aliquots of 100 µL of the standardized bacterial suspension were dispensed into the wells and incubated statically at 37 °C for 6, 12, 18, and 24 h, following the crystal violet-based procedure described by Negri et al. [30]. At each time point, the culture medium and non-adherent cells were removed, and the wells were gently washed with 0.05 M phosphate buffer at pH 6.5. The attached biofilm was fixed with 200 µL of 96% ethanol and subsequently stained with 200 µL of 0.1% crystal violet for 20 min. Excess stain was removed, and the crystal violet retained by the biofilm was solubilized with 33% acetic acid. Absorbance was measured at 590 nm using a microplate reader. The percentage inhibition of biofilm formation was calculated relative to the corresponding control using the predefined formula. The assay and data interpretation were conducted with consideration of the methodological recommendations for crystal violet-based biofilm quantification described by Andersen et al. [31].

### 2.9. Preclinical Toxicological Evaluation

The acute oral toxicity of *Bacillus subtilis* UzMU25 was evaluated in healthy, sexually mature white laboratory mice weighing 20 ± 2 g and male white laboratory rats weighing 160 ± 20 g. Before the experiment, all animals underwent a quarantine and acclimatization period of 10–14 days. The animals were allocated to six experimental groups, with 10 animals in each group, giving a total of 60 animals.

The tested bacterial preparation was administered directly into the stomach by oral gavage at doses of 2000, 4000, 5000, 6000, 8000, and 10,000 mg kg^−1^ body weight. Equal administration volumes were maintained among the experimental groups by adjusting the concentration of the tested preparation.

During the first 24 h after administration, the animals were observed hourly for changes in general condition, behavior, motor activity, tremors, respiratory abnormalities, and mortality. Thereafter, all animals were examined daily for 14 consecutive days. The monitored parameters included general clinical condition, activity, appearance and condition of the fur and skin, respiratory rate, food and water consumption, changes in body weight, and mortality.

The animals were maintained under standard laboratory animal-facility conditions and were provided with standard feed and drinking water *ad libitum*. The experimental procedures were conducted in accordance with the methodological recommendations described in the national guideline for preclinical evaluation of medicinal products [32].

At the end of the observation period, the median lethal dose, LD_50_, and the corresponding acute-toxicity classification were evaluated according to the method described in the following literature [33]. When no mortality occurred at the highest administered dose, the LD_50_ was considered to exceed 10,000 mg kg^−1^ body weight under the conditions of the present experiment.

### 2.10. Statistical Analysis

All experiments were performed in at least three independent replicates unless otherwise stated. Data are presented as mean ± standard deviation (SD). For the antagonistic-activity assay, differences in inhibition-zone diameters among the tested microorganisms were evaluated using one-way analysis of variance followed by Tukey’s multiple-comparison test. Corrected OD_590_ values obtained in the biofilm assay at 6, 12, 18, and 24 h were analysed using one-way analysis of variance followed by Tukey’s post hoc test. Data normality was assessed using the Shapiro–Wilk test, and homogeneity of variance was evaluated using Levene’s test. When the assumptions for parametric analysis were not met, the Kruskal–Wallis test followed by an appropriate multiple-comparison procedure was applied. Differences were considered statistically significant at *p* < 0.05. Statistical analyses were conducted using appropriate statistical software, and differences were considered significant at *p* < 0.05.

## 3. Results and Discussion

### 3.1. Isolation and Identification of Endophytic Bacteria from Inula helenium

Medicinal plants represent a valuable reservoir of endophytic microorganisms with potential applications in biotechnology, agriculture, and veterinary medicine. Due to their long-term association with host plants, endophytic bacteria frequently exhibit unique metabolic capabilities, including the production of hydrolytic enzymes, antimicrobial compounds, and growth-promoting metabolites. Consequently, the isolation of endophytes from medicinal plants has become an important strategy for discovering novel microorganisms with probiotic and biocontrol potential.

Fourteen cultivable bacterial isolates were recovered, including five from leaves, three from roots, two from flowers, and four from petioles. Because isolation frequencies were not normalised to the number of analysed tissue segments, these values are presented descriptively and were not used to infer statistically significant differences in endophyte abundance among plant tissues. The higher number of isolates obtained from leaf tissues may reflect tissue-specific differences in microbial colonization and nutrient availability within the host plant. Similar tissue-dependent distributions of endophytic bacteria have previously been reported in medicinal plants and are considered to result from variations in plant microenvironments and secondary metabolite composition.

The recovered isolates exhibited considerable diversity in colony morphology, pigmentation, surface characteristics, and hydrolytic activities (Table 1). Screening based on starch and casein degradation revealed substantial differences among isolates. Hydrolysis-zone diameters ranged from 1 to 10 mm, indicating varying capacities for extracellular enzyme production. Among all isolates, strain IH–B3 demonstrated the highest hydrolytic activity, producing the largest starch and casein degradation zones (8–10 mm) and displaying a characteristic rod-shaped morphology with rough, slightly convex colonies. The strong hydrolytic activity of this isolate suggests an enhanced ability to utilize complex substrates and may indicate an ecological advantage for colonization within plant tissues.

MALDI-TOF MS analysis assigned isolate IH-B3 to *Bacillus subtilis*, with an identification score of 2.26. According to the interpretive criteria applied by the MALDI-TOF system, this score was consistent with a reliable species-level database match. The selected isolate was subsequently designated *Bacillus subtilis* UzMU25. (see SI, Table S1). Members of the genus *Bacillus* are among the most frequently reported endophytic bacteria associated with medicinal plants and are recognized for their ability to produce a wide range of extracellular enzymes, antimicrobial metabolites, and probiotic factors. The identification of *B. subtilis* UzMU25 therefore provided a strong basis for subsequent investigations of its probiotic-related properties, antimicrobial activity, biofilm-forming capacity, and safety profile.

A 1444 bp 16S rRNA gene sequence was obtained from the selected isolate and deposited in the NCBI GenBank database under accession number PZ593820. The closest-reference sequence and corresponding percentage identity are being verified before final taxonomic interpretation.

**Figure 1:**
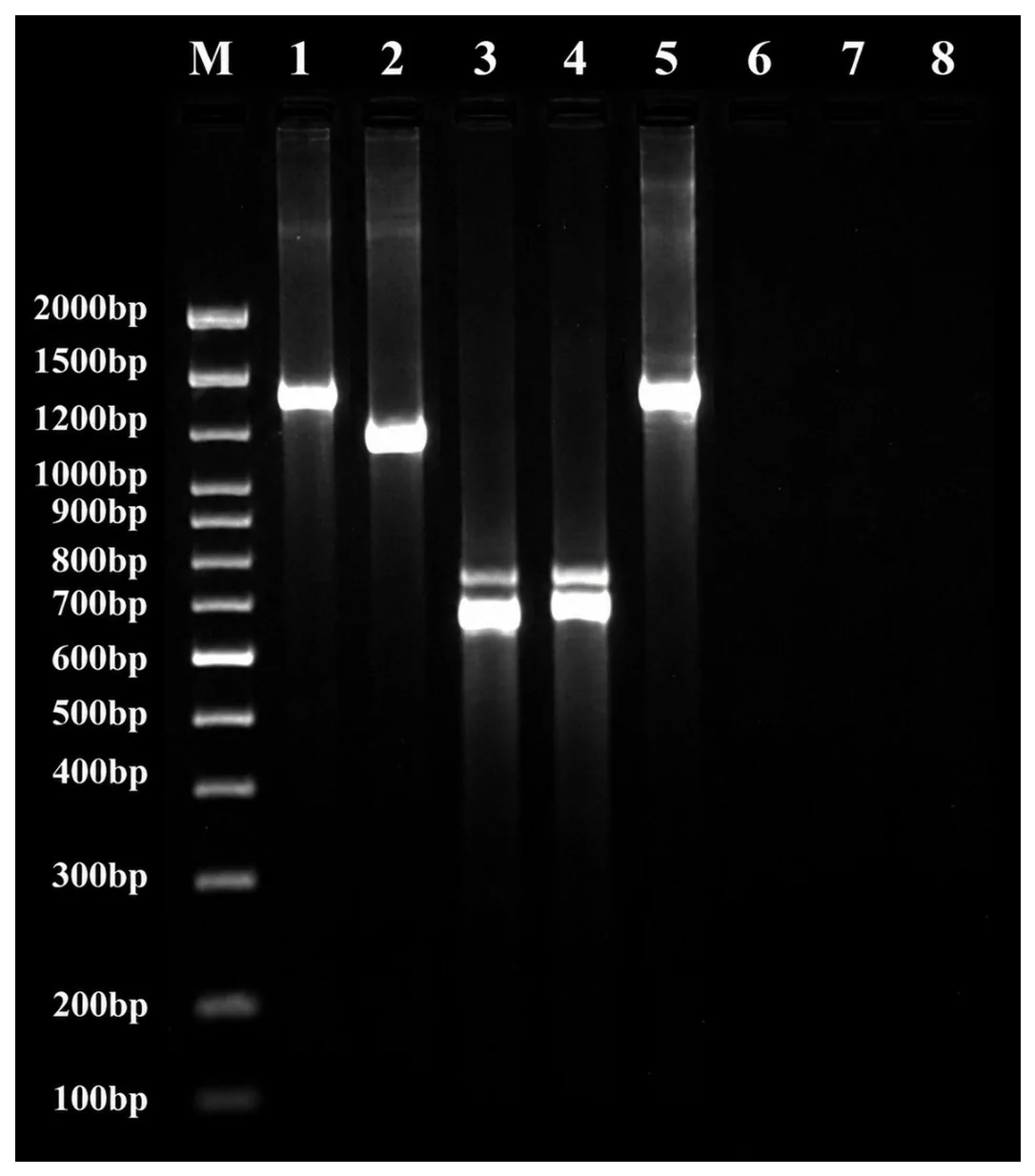
Agarose gel electrophoresis of PCR products targeting selected functional and toxin-associated loci in *Bacillus subtilis* UzMU25. Lanes: 1, *aprE*; 2, *sboA*; 3, *epsA*; 4, *levan*; 5, *groEL*; 6, *nheA*; 7, *hblA*; and 8, *cytK*. M, molecular-size marker.

PCR-based screening was performed to examine selected genetic loci associated with extracellular enzyme production, antimicrobial activity, biofilm-related functions, stress response, and toxin production in *Bacillus subtilis* UzMU25. Under the applied amplification conditions, PCR products were detected for *aprE*, *sboA*, *epsA*, *levan*, and *groEL* (Figure 1). The detection of *aprE* was consistent with the extracellular proteolytic activity observed in the phenotypic assays, because this locus is associated with the production of the extracellular serine protease subtilisin. The presence of *sboA*, a locus associated with subtilosin biosynthesis, may indicate the genetic capacity to produce a bacteriocin-like antimicrobial compound. However, the presence of this locus alone does not demonstrate gene expression or confirm that subtilosin was responsible for the antagonistic activity observed in the direct co-culture assays.

Amplicons corresponding to *epsA* and *levan* were also detected. These loci are associated with extracellular polysaccharide and levan biosynthesis, respectively, and may contribute to surface attachment, matrix formation, and adaptation to environmental conditions. Their detection is therefore consistent with the biofilm-forming phenotype observed for UzMU25. Nevertheless, a direct association between these loci and the measured biofilm biomass would require transcriptional analysis, gene-expression studies, or targeted mutational experiments.

The detection of *groEL*, which encodes a molecular chaperone involved in protein folding and cellular stress responses, may reflect the capacity of the strain to maintain protein homeostasis under environmental stress. However, because *groEL* is a widely conserved housekeeping gene, its presence should not be interpreted as a specific marker of probiotic activity. No PCR products were detected for the toxin-associated loci *nheA*, *hblA*, and *cytK* under the conditions used in this study. These genes are associated with non-hemolytic enterotoxin, hemolysin BL, and cytotoxin K, respectively. Their non-detection provides preliminary phenotypic–genetic support for the absence of these specific targets in UzMU25. However, targeted PCR screening is limited to the selected primer-binding regions and does not exclude the presence of sequence variants, other toxin-associated genes, or additional virulence determinants elsewhere in the genome.

Overall, the PCR profile was consistent with the presence of several selected functional loci and the non-detection of three toxin-associated targets. These findings complement the enzymatic and biofilm assays but should not be considered equivalent to whole-genome safety assessment. Whole-genome sequencing and comprehensive screening against validated virulence and antimicrobial-resistance databases are required before UzMU25 can be considered for practical probiotic, veterinary, or feed-related applications.

### 3.2. Probiotic-Related Biochemical Characteristics of B. subtilis UzMU25

The successful application of bacterial strains as probiotics requires the presence of several physiological and biochemical characteristics associated with safety, survival, and functional performance. Therefore, the probiotic-related properties of *B. subtilis UzMU25* were evaluated through enzymatic activity assays, hemolytic screening, and tolerance to gastrointestinal stress-associated factors (Table 2).

The investigated strain demonstrated positive catalase activity, as evidenced by rapid oxygen bubble formation following exposure to hydrogen peroxide. Catalase production is considered a beneficial probiotic trait because it enables bacteria to neutralize reactive oxygen species and withstand oxidative stress. In addition to protecting bacterial cells, catalase activity may contribute to maintaining intestinal redox balance and improving bacterial persistence under adverse environmental conditions. Similar catalase-positive phenotypes have been reported for numerous probiotic *Bacillus* strains and are considered advantageous for survival during gastrointestinal transit. The strain also exhibited lecithinase activity, indicating the ability to hydrolyze phospholipid-containing substrates. Extracellular phospholipase production may facilitate nutrient acquisition and enhance microbial adaptation to diverse ecological niches. Furthermore, lecithin degradation can contribute indirectly to the establishment of beneficial microbial communities through the release of assimilable metabolites. Although lecithinase activity is commonly detected in environmental *Bacillus* species, its biological significance in probiotic strains remains dependent on strain-specific characteristics and requires evaluation alongside additional safety indicators. Importantly, *B. subtilis* UzMU25 did not exhibit gelatinase activity or hemolytic activity. The absence of gelatinase activity suggests a reduced capacity to degrade host structural proteins, while the lack of hemolysis indicates that the strain does not damage erythrocytes under the tested conditions. These characteristics are widely regarded as essential safety criteria for probiotic candidate selection and support the non-pathogenic nature of the investigated isolate. Similar observations have been reported for commercially utilized probiotic *Bacillus* strains, where the absence of hemolytic and tissue-degrading activities is considered a prerequisite for safe application.

The strain further demonstrated tolerance to pH 6.5 and the presence of bile salts, indicating its ability to survive under conditions resembling those encountered within the gastrointestinal tract. Resistance to bile salts is particularly important because bile represents one of the primary physiological barriers limiting bacterial survival following oral administration. The observed tolerance therefore suggests that *B. subtilis* UzMU25 possesses physiological adaptations that may support its persistence within the digestive system.

Collectively, the biochemical characteristics observed in *B. subtilis* UzMU25 indicate a favorable combination of functional and safety-related traits. The presence of catalase and lecithinase activities, together with the absence of gelatinase and hemolytic activities and the demonstrated tolerance to bile salts and moderately acidic conditions, supports the potential of this strain as a promising candidate for further probiotic evaluation. Nevertheless, additional molecular safety assessments, including screening for virulence-associated and antibiotic resistance genes, would be valuable to further substantiate its suitability for practical applications.

### 3.3. Extracellular Hydrolytic Enzyme Activities of B. subtilis UzMU25

The production of extracellular hydrolytic enzymes is considered an important functional property of probiotic and biotechnologically valuable bacterial strains, as these enzymes contribute to substrate degradation, nutrient mobilization, and improved host digestion. In the present study, the enzymatic potential of *B. subtilis* UzMU25 was evaluated by determining its ability to produce amylase, protease, and lipase (Table 3).

**Figure 2:**
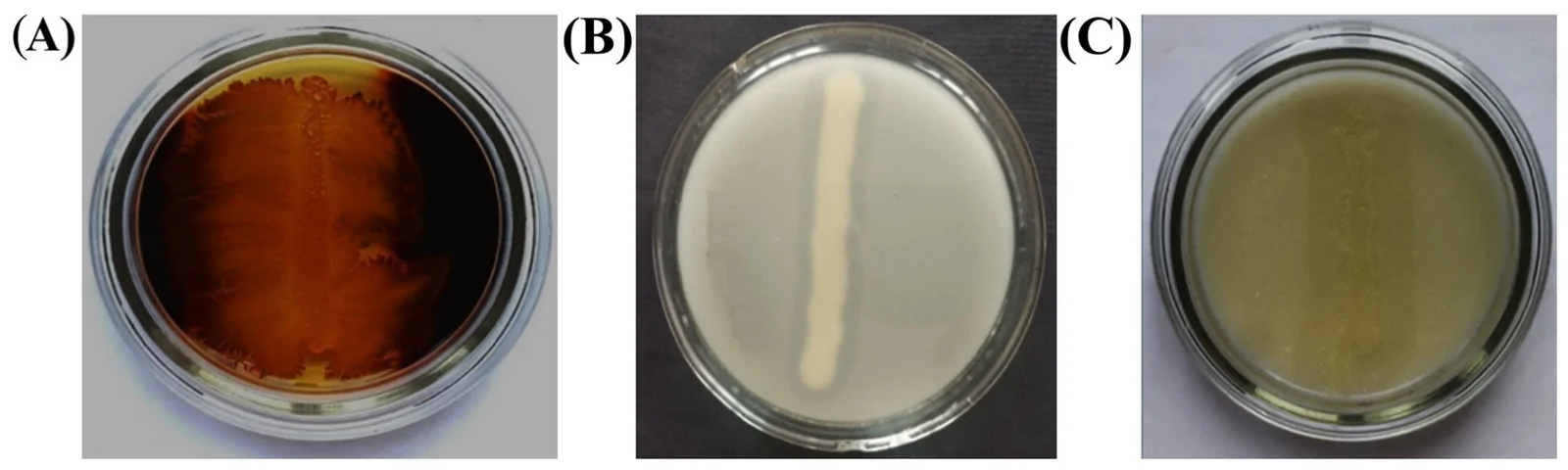
Amylase (**A**), lipase (**B**), and protease (**C**) enzyme activities of the *Bacillus subtilis* UzMU25 strain.

The strain demonstrated pronounced amylolytic activity, with starch-hydrolysis-zone diameters ranging from 46 to 52 mm (Figure 2A). This result indicates a strong ability to degrade complex polysaccharides and suggests that *B. subtilis* UzMU25 may contribute to the breakdown of carbohydrate-rich substrates. Amylase production is a particularly desirable trait for probiotic and feed-associated microorganisms because it may enhance starch digestion, improve nutrient availability, and support digestive efficiency in animals. Similar strong amylolytic activity has been reported for probiotic *Bacillus* strains used in agricultural and veterinary biotechnology, highlighting the importance of this enzymatic feature for practical application. In addition to amylase production, *B. subtilis* UzMU25 exhibited substantial proteolytic activity, with hydrolysis-zone diameters of 34–42 mm (Figure 2B). Protease-producing bacteria are of particular interest because proteolytic enzymes participate in the degradation of proteins into smaller peptides and amino acids, thereby improving nutrient accessibility. This property may be beneficial in probiotic formulations intended for animal nutrition, where efficient protein hydrolysis can support feed utilization and digestive performance. The relatively large proteolysis zones observed in the present study suggest that the strain possesses a well-developed extracellular protease system. Lipolytic activity was also detected, although to a lesser extent than amylase and protease production. The diameter of lipid hydrolysis zones ranged from 10 to 16 mm, indicating moderate lipase activity (Figure 2C). Lipases play an important role in the breakdown of lipid substrates and may contribute to improved fat digestion and assimilation. Although the lipolytic activity of *B. subtilis* UzMU25 was weaker than its amylolytic and proteolytic capacities, the presence of measurable lipase activity further supports the multifunctional enzymatic profile of the strain.

Overall, the enzymatic pattern observed in *B. subtilis* UzMU25 followed the order amylase > protease > lipase, indicating that carbohydrate and protein degradation represent the dominant extracellular activities of this isolate. Such a broad hydrolytic profile is advantageous from both ecological and applied perspectives. Within the plant endosphere, extracellular enzymes may facilitate nutrient acquisition and adaptation to plant-associated niches. From a biotechnological standpoint, the production of amylase, protease, and lipase supports the potential use of this strain as a probiotic or functional microbial additive in veterinary and agricultural systems. These findings are consistent with previous studies reporting that *Bacillus subtilis* strains frequently produce multiple extracellular enzymes, which contribute to their probiotic performance and industrial relevance.

### 3.4. Antibiotic Susceptibility of the B. subtilis UzMU25

Antibiotic susceptibility testing is an essential component in the evaluation of candidate probiotic strains, particularly when bacteria are intended for use in animal nutrition, veterinary biotechnology, or microbial biopreparation development. The presence of antibiotic resistance in probiotic candidates requires careful assessment because transferable resistance determinants may represent a potential biosafety concern. Therefore, the susceptibility profile of *B. subtilis* UzMU25 was evaluated against a panel of clinically relevant antibiotics, including imipenem, meropenem, ciprofloxacin, chloramphenicol, erythromycin, clindamycin, linezolid, norfloxacin, and vancomycin (Table 3).

**Figure 3:**
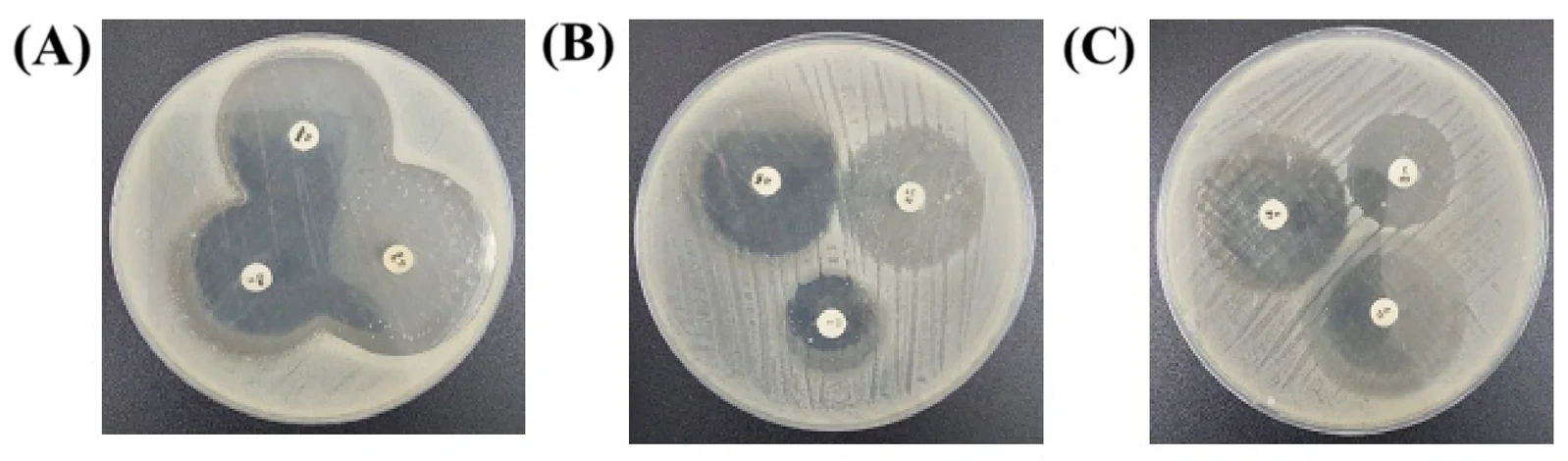
Antibiotic susceptibility of *Bacillus subtilis* UzMU25 determined by the disc-diffusion method. Within each panel, antibiotic discs are identified clockwise, beginning with the upper-left disc: (**A**) meropenem, imipenem, and ciprofloxacin; (**B**) norfloxacin, linezolid, and vancomycin; and (**C**) chloramphenicol, clindamycin, and erythromycin.

The results demonstrated that *B. subtilis* UzMU25 was susceptible to most of the tested antibiotics (Figure 3). The largest inhibition zone was observed for imipenem, reaching 30 mm, followed by meropenem with an inhibition zone of 25 mm (Figure 3A). The strain also showed susceptibility to norfloxacin, linezolid, erythromycin, and clindamycin with inhibition zones ranging from 17 to 24 mm (cf. Figure 3B,C). These findings suggest that the strain remains sensitive to several major antibiotic classes and does not display broad-spectrum antibiotic resistance under the tested conditions. Intermediate susceptibility was observed for ciprofloxacin and chloramphenicol, with inhibition zones of 23 and 24 mm, respectively (Figure 3A and Table 4). Such intermediate responses may reflect strain-specific differences in membrane permeability, efflux activity, or intrinsic tolerance mechanisms. However, intermediate susceptibility should be interpreted cautiously and should not be considered sufficient evidence of acquired resistance without further molecular analysis. Notably, *B. subtilis* UzMU25 displayed resistance to vancomycin, with an inhibition zone of 10 mm (Figure 3B). Vancomycin resistance or reduced susceptibility has been reported in some *Bacillus* species and may be associated with intrinsic cell wall-related characteristics. Nevertheless, from a probiotic safety perspective, this result requires further investigation to determine whether the observed resistance is intrinsic and non-transferable or associated with mobile genetic elements. Molecular screening for antibiotic resistance genes, particularly those associated with glycopeptide resistance, would therefore be important before practical application of the strain.

Overall, the antibiotic susceptibility profile of *B. subtilis* UzMU25 indicates that the strain is susceptible to several therapeutically important antibiotics, including carbapenems, macrolides, lincosamides, oxazolidinones, and fluoroquinolones. However, the observed vancomycin resistance and intermediate responses to selected antibiotics highlight the need for additional genomic safety assessment. Whole-genome sequencing or targeted PCR-based screening for antibiotic resistance determinants would strengthen the safety evaluation and provide stronger evidence supporting the potential use of *B. subtilis* UzMU25 as a probiotic or veterinary biotechnological candidate.

### 3.5. Antagonistic Activity of the B. subtilis UzMU25 Against Various Pathogenic Bacteria and Fungi

The ability of candidate probiotic strains to inhibit pathogenic microorganisms is one of the key functional criteria for their potential application in veterinary biotechnology, animal nutrition, and microbial biopreparation development. In the present study, the antagonistic activity of *B. subtilis* UzMU25 was evaluated against a panel of bacterial and fungal test organisms, including *Staphylococcus aureus*, *Pseudomonas aeruginosa*, *Escherichia coli*, *Serratia marcescens*, *Bacillus subtilis*, and *Candida albicans* (Table 5).

The results demonstrated that *B. subtilis* UzMU25 exhibited broad antagonistic activity against all tested microorganisms. The strongest inhibitory effect was observed against *Bacillus subtilis*, with an inhibition zone of 36 ± 0.23 mm, followed by *Escherichia coli* with 35 ± 0.20 mm. Considerable inhibition was also recorded against *Pseudomonas aeruginosa* and *Candida albicans*, with inhibition zones of 34 ± 0.24 mm for both microorganisms. The strain also inhibited *Staphylococcus aureus* and *Serratia marcescens*, producing inhibition zones of 30 ± 0.30 mm and 26 ± 0.22 mm, respectively. The observed inhibitory activity against both Gram-positive and Gram-negative bacteria suggests that *B. subtilis* UzMU25 may produce antimicrobial metabolites with a relatively broad spectrum of action. Members of the *Bacillus subtilis* group are known to synthesize diverse antimicrobial compounds, including lipopeptides, bacteriocin-like substances, organic acids and extracellular enzymes, which can interfere with pathogen growth, membrane integrity, and biofilm development. The strong inhibition of *E. coli* and *P. aeruginosa* is particularly relevant because these microorganisms are commonly associated with intestinal, environmental, and opportunistic infections in animals and humans. The antifungal activity observed against *Candida albicans* further supports the multifunctional antimicrobial potential of *B. subtilis* UzMU25. This result is important because probiotic strains capable of suppressing both bacterial and fungal opportunistic pathogens may provide broader protective effects in complex microbial communities. The inhibition of *C. albicans* may be associated with the production of antifungal lipopeptides or other diffusible metabolites; however, further chemical characterization is required to identify the active compounds responsible for this effect.

The antagonistic profile of *B. subtilis* UzMU25 indicates that the strain possesses strong inhibitory activity against a wide range of pathogenic and opportunistic microorganisms. These findings support its potential application as a probiotic candidate and biological control agent. Nevertheless, future studies should include positive antibiotic controls, cell-free supernatant assays, minimum inhibitory concentration determination and metabolite profiling to clarify the mechanisms underlying its antimicrobial activity.

### 3.6. Biofilm-Forming Ability of B. subtilis UzMU25

Biofilm formation is an important functional trait of probiotic and biotechnologically valuable bacterial strains because it may enhance environmental persistence, surface adhesion, colonization efficiency, and competitive exclusion of pathogenic microorganisms. In the present study, the biofilm-forming ability of *B. subtilis UzMU25* was evaluated using the crystal violet microtiter plate assay over a 24 h incubation period (Table 5). The negative control showed an optical density value of 0.09 ± 0.017, which was used as the critical cut-off value (ODc) for biofilm classification.

The corrected optical density values of *B. subtilis* UzMU25 were substantially higher than the control at all tested time points, confirming strong biofilm formation. During the first 6 h of incubation, the corrected OD value reached 0.537 ± 0.052, indicating rapid surface attachment and early biofilm development. The highest biofilm biomass was recorded at 12 h, with an OD value of 0.780 ± 0.025, suggesting active biofilm maturation during the early cultivation period (Table 5). At 18 h, the OD value remained high at 0.713 ± 0.027, while a slight decrease was observed at 24 h, reaching 0.627 ± 0.034. Nevertheless, the final value remained more than six times higher than the ODc, supporting the classification of the strain as a strong biofilm producer.

The strong biofilm-forming phenotype of *B. subtilis* UzMU25 may be beneficial for probiotic application, as biofilm formation can improve bacterial survival under unfavorable conditions and support prolonged persistence in complex microbial environments. In gastrointestinal or feed-associated systems, biofilm formation may contribute to colonization, resistance to environmental stress, and competition with undesirable microorganisms. Moreover, this property may support the strain’s antagonistic activity by facilitating stable establishment and localized production of antimicrobial metabolites. Visual observations also supported the quantitative results, as intense crystal violet staining and visible film-like structures were observed at the bottom of the experimental wells.

However, biofilm formation should be interpreted carefully from a safety perspective. While biofilm production can be advantageous for probiotic persistence, excessive or uncontrolled biofilm formation may raise concerns depending on the intended application. Therefore, the strong biofilm-forming ability of *B. subtilis* UzMU25 should be considered a potentially useful but strain-specific trait that requires further safety validation. Future studies should evaluate biofilm formation under gastrointestinal-like conditions, assess adhesion to intestinal epithelial models, and confirm the absence of virulence-associated biofilm traits through molecular or genomic analysis. Overall, these findings indicate that *B. subtilis* UzMU25 forms biofilms rapidly and maintains stable attached biomass over time, supporting its potential as a candidate probiotic and microbial biopreparation strain.

### 3.7. Preclinical Toxicological Safety Evaluations B. subtilis UzMU25 Probiotic Bacterial Strain

Preclinical safety evaluation is a critical requirement for bacterial strains intended for probiotic, veterinary, or feed-related applications. In the present study, the safety profile of *B. subtilis* UzMU25 was assessed through acute oral toxicity, dermal toxicity, cumulative toxicity, internal organ examination, and local irritation assays. These tests were conducted to determine whether the investigated strain caused mortality, behavioral changes, toxic accumulation, macroscopic pathological alterations, or irritation responses in laboratory animals under the tested experimental conditions.

In the acute oral toxicity assay, *B. subtilis* UzMU25 was administered to laboratory mice and rats at doses ranging from 1000 to 10,000 mg kg^−1^ body weight. During the 14-day observation period, no mortality was recorded in any experimental group. Animals receiving doses of 4000–6000 mg kg^−1^ showed no visible behavioral differences compared with intact animals, whereas temporary inhibition of activity was observed at higher tested doses. This effect lasted approximately 120–180 min and was reversible, with no subsequent mortality or severe clinical signs. In addition, topical application at 10 mL kg^−1^ did not produce toxic effects, redness, abnormal motor activity, fur changes, or mortality during the observation period. These findings indicate that *B. subtilis* UzMU25 has a low acute toxicity profile and can be classified as practically non-toxic, with an LD_50_ value exceeding 10,000 mg kg^−1^ under the tested conditions.

The cumulative toxicity of *B. subtilis* UzMU25 was further evaluated through repeated administration according to the dose-escalation scheme presented in Table 6. No animal deaths were observed throughout the experimental period, despite the progressive increase in administered doses up to the equivalent of 1.0 LD_50_. The absence of mortality after repeated exposure indicates that the strain did not exhibit cumulative toxic properties under the applied experimental conditions. Macroscopic examination after euthanasia revealed normal anatomical arrangement of internal organs and no free fluid accumulation in the pleural or abdominal cavities. The oral mucosa remained clean and moist, and no edema or hemorrhage was detected. The lungs, stomach, intestines, pancreas, kidneys, and adrenal glands showed no visible pathological alterations. The internal organ mass values presented in Table 7 are generally comparable between the control and treated groups, supporting the absence of pronounced toxic effects on major organs.

Local irritation was assessed using conjunctival and dermal irritation tests. In the conjunctival test, 0.5% and 5.0% solutions of *B. subtilis* UzMU25 culture liquid were applied to rabbit eyes, while distilled water served as the control. No redness, swelling, lacrimation, hyperemia, or delayed hypersensitivity reaction was observed after 15 min, 24 h, or 48 h. Similarly, repeated dermal application of the culture liquid to rat skin for 10 days did not cause irritation, redness, edema, inflammation, or other visible skin changes during the 14-day observation period. The reaction was scored as 0 points, confirming the absence of local irritation under the tested conditions. Taken together, the acute toxicity, cumulative toxicity, organ examination, conjunctival irritation, and dermal irritation results indicate that *B. subtilis* UzMU25 possesses a favorable preliminary safety profile. Nevertheless, before practical application, additional safety studies should include histopathological examination, hematological and biochemical blood analyses, genome-based screening for virulence factors and transferable antibiotic resistance genes, and controlled in vivo efficacy trials.

## 4. Conclusions

In this study, an endophytic bacterial strain isolated from the medicinal plant *Inula helenium* was identified as *Bacillus subtilis* and designated *B. subtilis UzMU25*. Among the fourteen recovered endophytic isolates, this strain was selected due to its superior cultural, morphological, and hydrolytic characteristics, particularly its strong activity toward starch and casein. The strain demonstrated several probiotic-related traits, including catalase and lecithinase activities, tolerance to pH 6.5 and bile conditions, and the absence of gelatinase and hemolytic activities. These properties indicate a favorable combination of functional and safety-associated characteristics.

The enzymatic profile of *B. subtilis* UzMU25 showed strong extracellular hydrolytic activity, with amylase, protease, and lipase production supporting its potential role in nutrient degradation and feed-associated applications. The strain also exhibited broad antagonistic activity against both bacterial and fungal microorganisms, including *Staphylococcus aureus*, *Pseudomonas aeruginosa*, *Escherichia coli*, *Serratia marcescens*, *Bacillus subtilis*, and *Candida albicans*. In addition, biofilm assays demonstrated that *B. subtilis* UzMU25 is a strong biofilm producer, with maximal biofilm formation observed during the early cultivation period. This property may contribute to environmental persistence, colonization capacity, and competitive exclusion of undesirable microorganisms.

The antibiotic susceptibility profile showed that *B. subtilis* UzMU25 remained susceptible to several clinically important antibiotics, including imipenem, meropenem, erythromycin, clindamycin, linezolid, and norfloxacin. However, resistance to vancomycin and intermediate responses to ciprofloxacin and chloramphenicol indicate that further molecular safety assessment is necessary. Preclinical toxicological studies demonstrated no mortality after acute oral administration up to 10,000 mg kg^−1^, no cumulative toxicity, and no detectable dermal or conjunctival irritation, supporting the preliminary safety of the strain under the tested conditions.

Overall, the results suggest that *B. subtilis* UzMU25 is a promising endophytic bacterial strain with probiotic-related, antagonistic, enzymatic, biofilm-forming, and preliminary safety characteristics. These findings support its further investigation as a candidate microorganism for veterinary biotechnology, animal feed supplementation, and microbial biopreparation development.

## Figures and Tables

**Table 1:** Morphological characteristics and preliminary starch- and casein-hydrolysis activities of bacterial isolates recovered from *Inula helenium*. (−, no visible hydrolysis zone; +, halo of X–Y mm; ++, halo of X–Y mm; +++, halo ≥ X mm.).

Colonies									
No	Name of Isolates	Shape	Size, mm	Hydrolysis Zone Size, mm	Color	Edge	Surface	Affinity for 1% Starch	Affinity for 1% Casein
1	*IH*–B1	rod	4–6	5–7	pure white	convex	wrinkled	++	++
2	*IH*–B2	spherical	1–2	2–3	Whitish	uneven	denser	+	+
3	*IH*–B3	rod-shaped	3–6	8–10	transparent pure white	rough	slightly convex	++	+++
4	*IH*–B4	rod-shaped	0.5–2	1–2	transparent milky	uneven	dense	+	+
5	*IH*–B5	rod-shaped	2–3	3–4	whitish	toothed	more convex	−	−
6	*IH*–I1	rod-shaped	2–4	3–6	cloudy whitish	toothed	convex	−	+
7	*IH*–I2	rod-shaped	2–4	4–6	yellowish	smooth	dense	++	+
8	*IH*–I3	rod-shaped	4–5	4–5	light yellowish	rough	convex	+	−
9	*IH*–G1	small rod	3–6	2–5	reddish	smooth	sticky	++	−
10	*IH*–G2	rod-shaped	1–2	3–4	yellowish	smoother	dense	+	−
11	*IH*–BB1	rod-shaped	2–3	3–4	milky	uneven	sticky	+	+
12	*IH*–BB2	small rod	2–6	1–4	milky	rough	dense, glossy	−	−
13	*IH*–BB3	rod	2–3	4–5	whitish	toothed	wrinkled	+	+
14	*IH*–BB4	large rod	1–2	2–5	milky	rough	sticky	+	+

**Table 2:** Biochemical and physiological characteristics of *B. subtilis* UzMU25. “+” indicates a positive reaction, detectable activity, or visible growth under the tested conditions; “−” indicates a negative reaction, absence of detectable activity, or no visible growth.

Study Strain	Catalase	Lecithinase	Gelatinase	Hemolysin	Acidity	pH	Horse Bile
*Bacillus subtilis*—UzMU25	+	+	−	−	90 °T	6.5	+

**Table 3:** Susceptibility of the *B. subtilis* UzMU25 to various concentrations of antibiotics.

Growth Inhibition Diameter, mm/Susceptibility of the Studied Strain to the Antibiotic					
No	Antibiotics	Antibiotic Content per Disk, mg	*Bacillus subtilis*—UzMU25		
			Resistant	Intermediate Resistant	Susceptible
1	Imipenem	10			30
2	Meropenem	10			25
3	Ciprofloxacin	5		23	
4	Levomycetin	5		24	
5	Erythromycin	15			24
6	Clindamycin	2			17
7	Linezolid	10			22
8	Norfloxacin	10			21
9	Vancomycin	5	10		

**Table 4:** Antagonistic activity of *B. subtilis* UzMU25 against bacterial and fungal test microorganisms. Inhibition zones are expressed as mean ± standard deviation (mm), based on three independent replicates (*n* = 3).

Tested Strains	Growth-Inhibition Zone, Zone Diameter, mm					
*B.subtilis*—UzMU25	*Staphylococcus aureus*	*Pseudomonas aeruginosa*	*Escherichia* *coli*	*Serratia marcescens*	*Bacillus subtilis*	*Candida albicans*
	30 ± 0.30	34 ± 0.24	35 ± 0.20	26 ± 0.22	36 ± 0.23	34 ± 0.24

**Table 5:** Results of the evaluation of biofilm formation by *B. subtilis* UzMU25.

	6 h			12 h			18 h			24 h		
A	0.259	0.152	0.121	0.273	0.512	0.309	0.456	0.431	0.266	0.325	0.366	0.41
B	0.188	0.252	0.1	0.222	0.264	0.412	0.285	0.166	0.378	0.373	0.242	0.296
C	0.192	0.106	0.132	0.296	0.541	0.345	0.386	0.381	0.214	0.366	0.28	0.6
D	0.386	0.368	0.249	0.239	0.439	0.458	0.393	0.278	0.112	0.289	0.313	0.192

**Table 6:** Cumulative toxicity dose-escalation scheme and mortality.

Days of Administration	Number of Animals (n = 10)	Share of LD_50_	LD_50_ ≥ 10,000 mg/kg
1	0/10	0.1	10,000
4	0/10	0.2	
8	0/10	0.4	
12	0/10	0.5	
16	0/10	0.6	
20	0/10	0.8	
24	0/10	1.0	

**Table 7:** Internal organ weights after oral administration of *B. subtilis* UzMU25 (M ± m; n = 10; *p* > 0.05).

Groups	Mass of Internal Organs, gr.								
	Liver	Kidneys	Spleen	Heart	Lungs	Stomach	Adrenal Glands	Thymus	Ovaries
Control	1.39 ± 0.05	0.45 ± 0.015	0.17 ± 0.035	0.21 ± 0.03	0.32 ± 0.015	0.36 ± 0.02	0.002 ± 0.0015	0.016 ± 0.002	0.11 ± 0.002
*Bacillus subtilis*—UzMU25	1.73 ± 0.07	0.48 ± 0.015	0.21 ± 0.03	0.28 ± 0.03	0.37 ± 0.015	0.42 ± 0.02	0.005 ± 0.002	0.021 ± 0.002	0.15 ± 0.002

## Data Availability

The raw data supporting the conclusions of this article will be made available by the authors on request.

## Supplementary Materials

The following supporting information can be downloaded at https://www.mdpi.com/article/10.3390/microorganisms14081819/s1, Table S1: MALDI-TOF MS identification of five isolates selected on the basis of preliminary hydrolytic activity; Table S2: Primers used for 16S rRNA gene amplification and targeted PCR screening of selected functional and toxin-associated loci in *Bacillus subtilis* UzMU25.

## References

[1] Hardoim P.R., Van Overbeek L.S., Berg G., Pirttilä A.M., Compant S., Campisano A., Döring M., Sessitsch A.J.M. (2015). The hidden world within plants: Ecological and evolutionary considerations for defining functioning of microbial endophytes. Microbiol. Mol. Biol. Rev..

[2] Compant S., Samad A., Faist H., Sessitsch A.J. (2019). A review on the plant microbiome: Ecology, functions, and emerging trends in microbial application. J. Adv. Res..

[3] Wu W., Chen W., Liu S., Wu J., Zhu Y., Qin L., Zhu B.J. (2021). Beneficial relationships between endophytic bacteria and medicinal plants. Front. Plant Sci..

[4] Dwibedi V., Rath S.K., Joshi M., Kaur R., Kaur G., Singh D., Kaur G., Kaur S.J. (2022). Microbial endophytes: Application towards sustainable agriculture and food security. Appl. Microbiol. Biotechnol..

[5] Singh M., Kumar A., Singh R., Pandey K.D.J.B. (2017). Endophytic bacteria: A new source of bioactive compounds. 3 Biotech.

[6] Adeleke B.S., Babalola O.O. (2021). Pharmacological potential of fungal endophytes associated with medicinal plants: A review. J. Fungi.

[7] Nguyen T.-D., Nguyen T.-T., Pham M.-N., Duong H.-N., Pham T.-T., Nguyen T.-P., Nguyen P.-T., Nguyen T.-U.T., Nguyen H.-H., Nguyen H.-T.J.R. (2023). Relationships between endophytic bacteria and medicinal plants on bioactive compounds production. Rhizosphere.

[8] Seal B.S., Drider D., Oakley B.B., Brüssow H., Bikard D., Rich J.O., Miller S., Devillard E., Kwan J., Bertin G.J.V.R. (2018). Microbial-derived products as potential new antimicrobials. Vet. Res..

[9] Hossain M.I., Sadekuzzaman M., Ha S.-D. (2017). Probiotics as potential alternative biocontrol agents in the agriculture and food industries: A review. Food Res. Int..

[10] Bhogoju S., Nahashon S.J.A. (2022). Recent advances in probiotic application in animal health and nutrition: A review. Agriculture.

[11] Kaur I.P., Chopra K., Saini A. (2002). Probiotics: Potential pharmaceutical applications. Agriculture.

[12] Hong H.A., Duc L.H., Cutting S.M. (2005). The use of bacterial spore formers as probiotics. Eur. J. Pharm. Sci..

[13] Todorov S.D., Ivanova I.V., Popov I., Weeks R., Chikindas M.L. (2022). Bacillus spore-forming probiotics: Benefits with concerns?. Crit. Rev. Microbiol..

[14] Poveda J., González-Andrés F. (2021). Bacillus as a source of phytohormones for use in agriculture. Appl. Microbiol. Biotechnol..

[15] Caulier S., Nannan C., Gillis A., Licciardi F., Bragard C., Mahillon J. (2019). Overview of the antimicrobial compounds produced by members of the Bacillus subtilis group. Front. Microbiol..

[16] Danilova I., Sharipova M. (2020). The practical potential of bacilli and their enzymes for industrial production. Front. Microbiol..

[17] Cossus L., Roux-Dalvai F., Kelly I., Nguyen T.T.A., Antoun H., Droit A., Tweddell R.J. (2021). Interactions with plant pathogens influence lipopeptides production and antimicrobial activity of Bacillus subtilis strain PTB185. Pestic. Biochem. Physiol..

[18] Bai X., Yi L., Zhao S., Xiu J., Li P., Shi R., Liang X., Zhang Y. (2025). Identification and comparative genomic analysis of two Bacillus subtilis producing antifungal lipopeptide. Pestic. Biochem. Physiol..

[19] Chun J., Park S.-M., Lee M., Ha I.J., Jeong M.-K.J.C. (2023). The sesquiterpene lactone-rich fraction of Inula helenium L. enhances the antitumor effect of anti-PD-1 antibody in colorectal cancer: Integrative phytochemical, transcriptomic, and experimental analyses. Cancers.

[20] Spiridon I., Nechita C.B., Niculaua M., Silion M., Armatu A., Teacă C.-A., Bodîrlău R. (2013). Antioxidant and chemical properties of Inula helenium root extracts. Open Chem..

[21] Altuntaş C., Gümrükçüoğlu A., Kalmuk N.A. (2026). Volatile organic compounds, phenolic content and antioxidant activities of Inula helenium subsp. orygalis and Inula orientalis flowers and leaves from Turkey. Plant Biosyst..

[22] Tiwari P., Thakkar S., Dufossé L. (2025). Antimicrobials from endophytes as novel therapeutics to counter drug-resistant pathogens. Crit. Rev. Biotechnol..

[23] Cui C., Liu X., Qiao G., Xia W., Ming H. (2026). Advances in the study of endophytic microorganisms in medicinal plants and their secondary metabolites. J. Appl. Microbiol..

[24] Xiang Y., Wei Z., Mo X., Liao Y., Wen C., Liao M., Wei J., Lin C., Li W., Chen J.J.F.M. (2026). Multidimensional analysis of pathogen epidemic patterns, their correlation with milk quality, and drug resistance and virulence traits of dominant pathogenic Escherichia coli in high-somatic-cell buffalo milk: Guangxi, China. Food Microbiol..

[25] Strobel G. (2003). Endophytes as sources of bioactive products. Microbes Infect..

[26] Leber A.L. (2020). Clinical Microbiology Procedures Handbook.

[27] Reiner K. (2010). Catalase Test Protocol.

[28] Volkova G.S., Serba E.M. (2021). New multistrain bacterial consortium for feed probiotics. Food Process. Tech. Technol..

[29] Lu Y., Fang C., Xiang L., Yin M., Qian L., Yan Y., Zhang L., Li Y. (2025). Characterization and therapeutic potential of three depolymerases against K54 capsular-type Klebsiella pneumoniae. Microorganisms.

[30] Negri M., Gonçalves V., Silva S., Henriques M., Azeredo J., Oliveira R. (2010). Crystal violet staining to quantify Candida adhesion to epithelial cells. Br. J. Biomed. Sci..

[31] Andersen J.B., Rybtke M., Tolker-Nielsen T. (2024). The dynamics of biofilm development and dispersal should be taken into account when quantifying biofilm via the crystal violet microtiter plate assay. Biofilm.

[32] Committee for the Update of the Guide for the Care and Use of Laboratory Animals, Institute for Laboratory Animal Research (1986). Guide for the Care and Use of Laboratory Animals.

[33] Berezovskaya I.V. (2003). Classification of Substances with Respect to Acute Toxicity for Parenteral Administration. Pharm. Chem. J..

