# Supplementary material for: Probiotic and Antimicrobial Potential of Endophytic B. subtilis UzMU25 Isolated from Inula helenium
Source: Microorganisms. 2026 Aug 18;14(8):1819. doi: 10.3390/microorganisms14081819 (PMC13515722; doi:10.3390/microorganisms14081819)
Supplement: Supplementary file 1 [file microorganisms-14-01819-s001.zip › microorganisms-4381441-supplementary.pdf]

Supplementary material

Probiotic and Antimicrobial Potential of Endophytic *B. subtilis* UzMU25 Isolated from *Inula helenium*

Luiza Tagayeva[1], Kunduz Normurodova[1], Shermat Jabborov[2,*], Bobur Khasanov[2] and Jamoliddin Razzokov[3,4,5]

[1]Department of Biotechnology and Microbiology, Faculty of Biology and Ecology, National University of Uzbekistan, University Street 4, Tashkent 100174, Uzbekistan
[2]Scientific Research Institute of Livestock and Poultry, Tashkent, Kibray District, Qizil Shalola Village, Tashkent 111121, Uzbekistan
[3]Institute of Fundamental and Applied Research, National Research University TIIAME, Tashkent 100000, Uzbekistan
[4]Department of Natural Sciences, Karshi State Technical University, Mustaqillik Avenue Street 225, Karshi 180100, Uzbekistan
[5]Department of Biotechnology, Tashkent State Technical University, Universitet 2, Tashkent 100095, Uzbekistan
*Correspondence: jabborovshermat4@gmail.com

Table S1: MALDI-TOF MS identification of five isolates selected on the basis of preliminary hydrolytic activity.

Isolate	Best database match	MALDI-TOF score	Interpretation
IH-B1	*Bacillus subtilis*	2.11	Reliable species-level match
IH-B3	*Bacillus subtilis*	2.26	Reliable species-level match
IH-I2	*Klebsiella oxytoca*	2.07	Reliable species-level match
IH-G2	*Pseudarthrobacter oxydans*	2.09	Reliable species-level match
IH-BB1	*Bacillus amyloliquefaciens*	2.17	Reliable species-level match

Genomic DNA extracted from the pure culture of *Bacillus subtilis* UzMU25 was evaluated using a NanoDrop™ Eight Spectrophotometer (Thermo Fisher Scientific, USA). DNA concentration was recorded in ng μL^{-1} based on absorbance at 260 nm, while DNA purity was assessed using the A260/A280 absorbance ratio. Only DNA preparations of adequate concentration and purity were used for subsequent PCR amplification.

The nearly full-length bacterial 16S rRNA gene was amplified using the universal primer pair 27F/1492R. Additional primer pairs were used to screen selected functional and toxin-associated loci in UzMU25. Reference sequences for each target were aligned using ClustalW, and primers were designed from conserved sequence regions. The selected functional targets included *aprE*, associated with extracellular serine-protease production; *sboA*, associated with subtilosin biosynthesis; *epsA*, associated with extracellular-polysaccharide formation; *levan*, associated with levan biosynthesis; and *groEL*, encoding a conserved molecular chaperone involved in protein folding and stress response. The toxin-associated targets included *nheA*, *hblA*, and *cytK*, associated with non-haemolytic enterotoxin, haemolysin BL, and cytotoxin K, respectively.

PCR products were separated by agarose-gel electrophoresis and visualised using an appropriate nucleic-acid staining system. The presence of an amplification product of the expected size was recorded as target detection. Lack of amplification was reported as non-detection under the applied PCR conditions rather than definitive absence of the corresponding locus from the genome.

Table S2. Primers used for 16S rRNA gene amplification and targeted PCR screening of selected functional and toxin-associated loci in *Bacillus subtilis* UzMU25

Target	Primer	Nucleotide sequence, 5′–3′	Functional category
16S rRNA	16S_27F	AGAGTTTGATCCTGGCTCAG	Taxonomic identification
16S rRNA	16S_1492R	GGTTACCTTGTTACGACTT	Taxonomic identification
aprE	aprE_F	CCGTGAGAAGCAAAAAATTGTGGATCA	Extracellular protease-associated locus
aprE	aprE_R	ATTTATTGTGCAGCTGCTTGTACGTTG	Extracellular protease-associated locus
sboA	sboA_F	CATCCTCGATCACAGACTTCACATG	Subtilosin-biosynthesis-associated locus
sboA	sboA_R	CGCGCAAGTAGTCGATTTCTAACAC	Subtilosin-biosynthesis-associated locus
epsA	epsA_F	ATGAATGAGAATATGAGTTTCAAAGAA	Exopolysaccharide-associated locus
epsA	epsA_R	CTCCCCGAAATGTTTTATCC	Exopolysaccharide-associated locus
levan	levan_F	CGTACGGCGTCTCTCATATC	Levan-biosynthesis-associated locus
levan	levan_R	CCTTGTTCAAGGATGCTGTT	Levan-biosynthesis-associated locus
groEL	groEL_F	ATGGCAAAAGAAATTAAGTTTAGTGA	Stress-response and protein-folding locus

groEL	groEL_R	TTACATCATTCCGCCCATAC	Stress-response and protein-folding locus
nheA	nheA_F	GTTAGGATCACARTCACCGC	Toxin-associated locus
nheA	nheA_R	ACGAATGTAATTTGAGTCGC	Toxin-associated locus
hblA	hblA_F	AAGCAATGGAATACAATGGG	Toxin-associated locus
hblA	hblA_R	AGAATCTAAATCATGCCACTGC	Toxin-associated locus
cytK	cytK_F	ACAGATATCGGKCAAAATGC	Toxin-associated locus
cytK	cytK_R	TCCAACCCAGTTWSCAGTTCD	Toxin-associated locus
